# P22 – Set-up and validation of a mouse model for food-allergy induced atopic dermatitis

**DOI:** 10.1186/2045-7022-4-S1-P77

**Published:** 2014-02-28

**Authors:** Anneke Rijnierse, Desiree Veening, Tjalling Wehkamp, Prescilla Jeurink, Johan Garssen, Leon Knippels

**Affiliations:** 1 Danone Research, Centre for Specialised Nutrition, The Netherlands; 2 Utrecht Institute for Pharmaceutical Sciences, Utrecht, The Netherlands

## 

Atopic dermatitis (AD) is a complex disorder characterized by flares of red and itchy skin. Infants with AD often also suffer from food allergy. Both ingestion of and skin contact with the antigen can exacerbate the AD. The affected skin is characterized by oedema formation and shows typical Th2 responses by increased levels of IL-4 and IL-13, but not IFN-γ. Furthermore, there is an influx of eosinophils and CD4+ cells. The CD4+ cells are previously described to originate from the gut mucosa where they developed upon oral sensitization to the food allergen.

From both specific oligosaccharides and TLR ligands, it is clinically demonstrated that it can affect the development of AD, by skewing the immune response away from an allergic phenotype.

In this study, a mouse model to study the effect of dietary compounds on the development and treatment of food allergy-induced AD will be set-up. For this model, BALB/c mice are orally sensitized with ovalbumin (OVA; generally used to induce hen’s egg allergy) plus cholera toxin adjuvant once a week, for 5 consecutive weeks. One week after the last sensitization, the mouse skin will be challenged with OVA on tape-stripped back skin. This is repeated 3 days later, and again 4 days later, the mice are sacrificed. Outcome parameters as skin thickness, inflammatory cell influx and skin mucosal cytokine levels will be studied. Following development of the AD model the effect of dietary intenvention with a combination of specific OS and TLR ligands will be evaluated on the different outcome parameters.

